# Draft Genome Sequence of a *Staphylococcus warneri* Strain Isolated from a Preterm Neonate Blood Sepsis Patient at the Royal Infirmary, Edinburgh, Scotland

**DOI:** 10.1128/genomeA.00877-14

**Published:** 2014-09-04

**Authors:** K. A. Kropp, A. Lucid, J. Carroll, V. Belgrudov, P. Walsh, B. Kelly, C. Smith, P. Dickinson, A. O’Driscoll, K. Templeton, P. Ghazal, R. D. Sleator

**Affiliations:** a Department of Biological Sciences, Cork Institute of Technology, Bishopstown, Cork, Ireland; b Nsilico, Cork, Ireland; c Department of Computing, Cork Institute of Technology, Bishopstown, Cork, Ireland; d Division of Pathway Medicine, University of Edinburgh, Edinburgh, United Kingdom; e Microbiological Diagnostic Unit, Royal Infirmary, University of Edinburgh, Edinburgh, United Kingdom

## Abstract

Herein, we report the draft genome sequence of *Staphylococcus warneri* ED-NGS-1001, cultivated from a blood sample taken from a preterm neonate blood sepsis patient at the Royal Infirmary, Edinburgh, Scotland, United Kingdom.

## GENOME ANNOUNCEMENT

*Staphylococcus warneri* is a Gram-positive, clinically important pathogen and a leading cause of blood sepsis (1–3). Preterm neonates are a particularly highly susceptible group, due to their immature immune status and the invasive procedures they are subjected to in neonatal intensive care unit (ICU) settings (3–5). Rapid detection of blood sepsis and characterization of the causative pathogen are critical to enable proper treatment (6–8). In the ClouDx-i project we aim to extend knowledge of currently circulating pathogens linked with neonatal blood sepsis to inform the development of improved molecular diagnostic assays. Herein, we present the draft genome of a *Staphylococcus warneri* strain, isolated from a preterm neonate.

The isolate was grown overnight at 37°C on Luria broth (LB) agar, and genomic DNA was isolated using Qiagen genomic tips (Venlo, Limburg, Netherlands). Genomic DNA fragments were produced ranging in size from 2 to 10 kb using sonication. Fragments were subsequently used to produce a non-size-selected genome library using the Nextera mate pair kit (Illumina, San Diego, CA). Resulting libraries were sequenced on an Illumina MiSeq using a MiSeq reagent kit (v. 3). Genomic sequence assembly, analysis, and automated reporting were achieved using Simplicity (9). This produced 1,795,954 total reads, resulting in an estimated 142-fold coverage of the genome. The average G+C content was 32.62%. Sequence assembly was performed using a *de novo* assembly pipeline based on the Spades 3.10 assembly tool with k-mers K21, K33, K55, K77, K99, and K127 nucleotides in length, resulting in a total of 69 contigs, of which 12 were >1,000 bp, representing 98.98% of total sequence information, with the largest contig being 1,239,736 bp. Postassembly processing was performed by Spades and only scaffolds of length greater than 1,000 bp were considered when creating a draft genome, resulting in a molecule of 2,517,955 bp. We initially annotated the genome with Prokka (10) and used the identified 16S rRNA gene to confirm the species as *Staphylococcus warneri*. A scaffold of the genome was produced with Contiguator2 and we attempted to identify the closest related strain by BLASTing the scaffold against the NCBI database, returning the strain *S. warneri* SG1 as a closely related but not identical strain. The genome was then screened using the Glimmer 3 tool (11), identifying 2,396 open reading frames (ORFs). Predicted ORFs were compared to the Uniprot Trembl database (12) using BLASTp. A total of 1,321 ORFs were mapped to the protein database and potential virulence factors were identified by comparison to a local database built from the VFDB (13) and Victors databases with BLASTp. A 75% amino acid sequence identity cutoff was employed while considering only alignments longer than 100 amino acids, identifying 22 hits, including the MecA ORF, and identifying ED-NGS1001 as a methicillin-resistant *Staphylococcus aureus* (MRSA) strain.

Samples were handled in accordance with local ethical approval by the ethics committees of the NHS Lothian SAHSC Bioresource and NHS R&D office (project identification [ID] 2011/R/NE/01) and the HSS BioResource (RequestID 13/ES/0126).

### Nucleotide sequence accession numbers.

This whole-genome shotgun project has been deposited at DDBJ/EMBL/GenBank under the accession number JPOW00000000. The version described in this paper is version JPOW01000000.
